# Potential of Mushroom Compounds as Immunomodulators in Cancer Immunotherapy: A Review

**DOI:** 10.1155/2018/7271509

**Published:** 2018-04-22

**Authors:** Peter Amwoga Ayeka

**Affiliations:** Department of Biological Sciences, Faculty of Science, Egerton University, P.O. Box 536-20115, Egerton, Kenya

## Abstract

Since time immemorial, plants and their compounds have been used in the treatment and management of various ailments. Currently, most of conventional drugs used for treatment of diseases are either directly or indirectly obtained from plant sources. The fungal group of plants is of significance, which not only provides food directly to man but also has been source of important drugs. For instance, commonly used antibiotics are derived from fungi. Fungi have also been utilized in the food industry, baking, and alcohol production. Apart from the economic importance of the microfungi, macrofungi have been utilized directly as food, which is usually got from their fruiting bodies, commonly known as mushrooms. Due to their richness in proteins, minerals, and other nutrients, mushrooms have also been associated with boosting the immune system. This makes mushrooms an important food source, especially for vegetarians and immunosuppressed individuals including the HIV/AIDS persons. In complementary and alternative medicines (CAMs), mushrooms are increasingly being accepted for treatment of various diseases. Mushrooms have been shown to have the ability to stimulate the immune system, modulate humoral and cellular immunity, and potentiate antimutagenic and antitumorigenic activity, as well as rejuvenating the immune system weakened by radiotherapy and chemotherapy in cancer treatment. This potential of mushrooms, therefore, qualifies them as candidates for immunomodulation and immunotherapy in cancer and other diseases' treatment. However, a critical review on mushroom's immune modulating potential in cancer has not been sufficiently addressed. This review puts forward insights into the immune activities of mushroom associated with anticancer activities.

## 1. Introduction

Humanity continues to suffer the scourge of cancer, a disease that is associated with uncontrolled cell growth. In 2013, it was reported to be among the leading causes of death, second to cardiovascular diseases. It is estimated that death due to cancer will rise to thirteen million in 2030 [1–3]. The fight against cancer has intensified in the past decades with multidirectional approach including behavioral and dietary change, chemotherapy, radiotherapy, surgery, and recently immunotherapy. Unfortunately, these approaches are not void of serious side effects spanning from recurrence and weakened immune system to reduced quality of life (QoL) of patients. This has raffled scientists, leading to concerted efforts of finding better therapies that, apart from managing the cancerous cells, boost the immune system to fight cancer and other diseases [4]. Among these therapies, complementary and alternative medicine (CAM) has been fronted as an alternative due to its potential of holistic treatment including augmenting the immune system. Many CAMs are plant-derived, including algae and mushrooms that have been used widely in many parts of the world, where they are regarded as biological response modifiers (BRMs) and immunoceuticals [5]. Mushrooms are the spore-producing reproductive structures of fungi. Ancient classification placed fungi in plant kingdom, but current classification recognizes fungi as an independent group of organisms under the kingdom Mycota, basically due to possession of chitin within their cell walls. Mushrooms are the fleshy, spore-bearing fruiting body of a fungus, typically produced above ground on soil or on its substrate, mainly by the Basidiomycota and Ascomycota group. Although in wild mushrooms are seasonal and can be collected and used, they can be domesticated through spore or tissue culture in the laboratories. There are over 14,000 mushroom species but only about 3000 are edible, with approximately 700 exhibiting medicinal properties and 1% being poisonous [6]. For many years, mushrooms have been associated with nutritional and medicinal properties including immune modulation and antitumor properties [6–11]. Edible mushrooms, according to research, are believed to strengthen the immune system by exerting their effects on cellular activities, secondary production of chemical compounds that boost the immune system, and helping treat diseases and restore cell immunity destroyed by radiation and chemotherapy, and this is linked majorly to *β*-glucans [12, 13].

A key, frequently reported protective mechanism exerted by mushrooms against cancer is the capacity to stimulate the immune system response, where beta-glucan, a water-soluble polysaccharide, activates immune cells and proteins and macrophages, T cells, natural killer cells, and cytokines that attack tumor cells [10]. White button mushroom* Agaricus bisporus* is an example of dietary mushrooms; apart from having bioactive antioxidants and anticarcinogenic substances, these bioactive compounds also alter aromatase enzyme activity. This enzyme is involved in the conversion of androgens to proliferative estrogenic intermediates which are closely linked to breast cancer development [14, 15]. Furthermore, nonpolysaccharide constituents in species like Shiitake and Oyster mushrooms have biological activity against murine skin cancer and human prostate carcinoma cells [16]. The antitumor and immunomodulation activity of mushroom is exhibited by both crude fungal extracts and pure compounds. The polysaccharide fraction that is mainly composed of *β*-glucans present in the cell walls is responsible for immune modulating effects in a number of ways including activating phagocytic activity and production of reactive oxygen intermediates, inflammatory mediators, and cytokines production [7, 10, 17].

## 2. Selected Medicinal Mushrooms and Their Anticancer Activity

Mushrooms can either be edible, medicinal, or poisonous. Many mushroom species, either edible or poisonous, contain bioactive compounds that are of significance to human health.

Mushroom cell walls contain two important compounds, chitin and *β*-glucans. Of these two, *β*-glucans *β*(1→3), *β*(1→4), and *β*(1→6) make mushroom of significance in health and treatment of various diseases [18–20]. In addition to these compounds, there are other important components in mushrooms. They include polysaccharides, polysaccharide-protein complexes, agaritine, ergosterol, selenium, polyphenols, and terpenoids. Apart from therapeutic properties associated with these compounds, they are generally regarded as biological response modifiers (BRMs). Both* in vitro* and* in vivo* experiments support the therapeutic activities of mushroom compounds. These compounds modulate the immune system to fight tumors and other diseases. These include augmenting the immune system through stimulating lymphocytes, NK cells, and macrophages, enhancing cytokine production, inhibiting proliferation of cancer cells, promoting apoptosis, and blocking angiogenesis, in addition to being cytotoxic to cancer cells [21, 22]. These compounds come in contact with intestinal cells, the frontline of intestinal immune system which interacts with the antigens, thereby playing a role in intestinal immune response and inducing inflammatory response if necessary [23]. Mushroom-derived polysaccharides and polysaccharide-protein complexes are considered as one of the major sources of therapeutic agents for immunomodulatory and antitumor properties [21, 24]. More than 50 mushroom species have yielded potential immunoceuticals with immunomodulatory and antitumor effects* in vitro* and* in vivo* and also in human cancers. They include lectins, polysaccharides, polysaccharides-peptides, polysaccharide-protein complexes like lentinan, schizophyllan, polysaccharide-K, polysaccharide P, active hexose correlated compounds (AHCC), and Maitake D fraction. These compounds are derived from* Ganoderma lucidum, G. tsugae, Schizophyllum commune*,* Sparassis crispa, Pleurotus tuber-regium*,* P. rhinoceros, Trametes robiniophila *Murill,* Coriolus versicolor, Lentinus edodes, Grifola frondosa, *and* Flammulina velutipes*, among others [17]. These mushrooms are associated with the treatment of various cancers including breast, colorectal, cervical, skin, liver, ovarian, bladder, prostate, gastric, skin, lung, leukemia, and stomach cancers (Table 1). Mushroom compounds utilize different mechanisms to modulate immunity system in cancer treatment. For instance, water extracts of* Agaricus blazei* Murill (AbM) fruiting bodies induce production of TNF-alpha, IL-8, and NO− [25]; it is low molecular weight polysaccharides that suppress tumor growth and angiogenesis* in vivo* [26], and they contain agaritine and ergosterol which are capable of inducing apoptosis in leukemia cells and inhibit tumor-induced angiogenesis [27, 28].* Ganoderma lucidum* polysaccharides and triterpenoids are potent inhibitors of tumor growth* in vitro* and* in vivo* [14]. Furthermore, extracts of* G. lucidum* and* G. tsugae* are able to inhibit growth of colorectal cancer cells* in vitro* [29]. Schizophyllan, from* Schizophyllan commune*, a *β*(1–3) and *β*(1–6) D-glucan, is less effective against gastric cancer but increases survival of patients with head and neck cancer. In cervical cancer, it prolongs survival and time to recurrence for stage II, and it is more effective when injected directly to the cancer mass [30], suggesting a direct cytotoxicity effect to tumor cells. There is also a remarked increase in monocytes and granulocytes in blood and spleen, leading to production of IL- 6 and IL- 8 after use of Cauliflower mushroom* (Sparassis crispa)*, suggesting that it has immunomodulatory properties [31]. Other mushrooms like* P. tuber-regium* and* P. rhinoceros* polysaccharides have antitumor effects, where they are able to induce expression and proliferation of NK cells, macrophages, and T helper cells in mice [32–34] and* Trametes robiniophila *Murrill (Huaier), an officinal fungus in China, has been applied in TCM for approximately 1600 years [35] and its proteoglycans display apoptosis, antiangiogenesis, drug resistance reversal, antimetastasis, and system immune activation. Table 1 highlights selected mushrooms studied in various cancers.

## 3. Mechanism of Modulating the Immune System by Anticancer Mushroom Compounds

**Figure 1:**
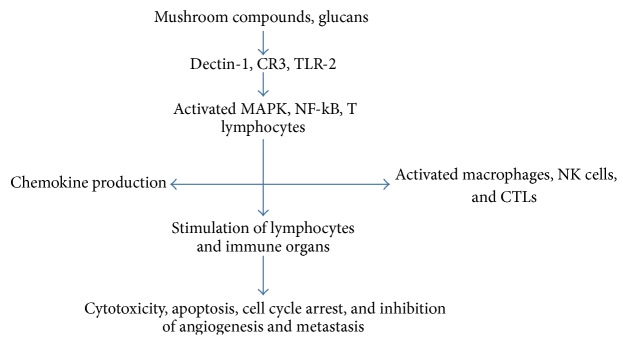
Probable immunomodulation mechanism of action of mushroom glucans. They utilize Dectin-1, CR3, and TLR-2 leading to activation and signal transduction of T lymphocytes, MAPK, and NF-kB, in turn leading to chemokine production and activation and stimulation of lymphocytes, macrophages, and NK cells, which results in inhibition of cancer proliferation through either direct toxicity, apoptosis, and cancer cell cycle arrest or hindering angiogenesis and metastasis of cancer cells.

Mushroom compounds are known to fight cancers through modulating both innate (nonspecific) and adaptive (specific) immune systems. The response of an immune system after invasion by antigens heavily relies on interaction between host pattern recognition receptors (PRRs) and pathogen associated molecular patterns (PAMPs). PRRs initiate innate immunity through pathogen recognition, while toll-like receptors (TLRs) initiate signaling pathways that coordinate innate immunity and trigger adaptive immunity against various pathogens [56]. Mushroom cell walls have compounds, especially *β*-glucans, which are thought to be a major PAMP involved in initiating an immune response. The receptors of *β*-glucans, Dectin-1, are expressed on dendritic cells, macrophages, neutrophils, and monocytes [57, 58]. Binding of Dectin-1 and *β*-glucans leads to signal transduction which in turn activates T cells, mitogen activated protein kinases (MAPK), and nuclear factor kappa B (NF-kB), resulting in cytokine production [59, 60]. More so, mushroom compounds are recognized by the PRR, by utilizing the Dectin-1, toll-like receptor 2 (TLR-2), and the complement receptor 3 (CR3). PAMP binds to TLR2 initiating the adaptive immunity and PAMP-PRR on monocytes, dendritic cells, granulocytes, and NK cells of the innate immune system [61–65] leading to activation of immune cells, cytokine production, and expression of adhesion molecules [66, 67], as illustrated in Figure 1.

In addition, glucans, which are pharmacologically important compounds of mushrooms, are resistant to acid and therefore they are able to pass through the stomach to the duodenum, where they interact with receptors, activating them to produce lysozyme, reactive oxygen radicals, and nitrogen oxides. These in turn stimulate the production of cytokines that activate phagocytes and leukocytes, leading to local or systemic immunity [68–70].

The efficiency of beta-glucans to activate leukocytes is dependent not only on their conformation but also on solubility in water, molecular weight, and degree of substitution and branching. Their pharmacological activity can be linked to interaction with specific *β*-glucopyranose receptors on leukocytes. This interaction activates leukocytes, which in turn stimulate phagocytosis, cytotoxicity, and production of cytokines by leukocytes [71, 72].

## 4. Effects of Mushroom Compounds on Cytokine Production

Mushroom compounds exert their immune modulating properties through a variety of molecular mechanisms. Some upregulate genes which leads to production of anti-inflammatory and anticancer cytokines. Studies with mushroom compounds have shown that a number of genes and cytokines are variously affected following* in vitro* and* in vivo* treatments. Cytokines are the messengers of the immune system and are either proteins or glycoproteins, secreted by immune cells, to regulate innate and adaptive immune system [6]. Following an oral uptake of mushrooms/mushroom compounds, intestinal immune factors are activated, that is, dendritic cells and macrophages that secrete cytokines that initiate local or systemic immunity. Intestinal epithelial cells are also stimulated to secrete IL-7, an important cytokine in cancer immunotherapy [73, 74].

Incubation of promonocytic THP-1 cells with* Agaricus blazei *Murill extract upregulates many genes that are associated with anticancer chemokines, leading to secretion of a number of cytokines such as IL-23*α* subunit in the IL-12 family, IL-1*β*, monocyte chemoattractant protein-1 (MCP-1), granulocyte colony stimulating factor (G-CSF), and tumor necrosis factor-alpha (TNF-*α*) [27, 36]. Furthermore, Volman et al. [75] showed that* Agaricus bisporus* fruit bodies, caps, and stipe increase TNF-*α* production by bone marrow derived macrophages (BMM).

*Ganoderma lucidum*, on the other hand, is longevity-promoting tonic herb and the biological activities, especially antitumor and immunomodulatory properties, include stimulating T cells and inflammatory response by expression and production of chemokines including IL-1, IL-2, IL-6, TNF-*α*, and interferon-gamma (IFN-*γ*) [4, 41, 42]. Grifolan from* Grifola frondosa* promotes macrophage activities by increasing IL-1, IL-6, and IL-8 production, ultimately activating and increasing the number of leukocytes [45–47, 76]. Other compounds from mushrooms such as polysaccharide peptide (PSP), polysaccharide (PSK), and lentinan provoke* in vitro* secretion of varied cytokines, namely, IL-1, IL-2, IL-6, IL-8, TNF, and interferons [49].

In addition, Bittencourt et al. [52] demonstrated that *α*-glucan from* Pseudallescheria boydii* stimulates* in vitro* secretion of TNF-*α* and IL-12. The increased secretion of IL-12 indicates a polarization of naïve T cells into T helper (Th) type 1 skewed responses which are important in fighting cancer cells [37, 77]. The extract from* Sparassis crispa* stimulates splenocytes to secrete cytokines in mice and this is triggered by granulocyte macrophage colony stimulating factor (GM-CSF) and Dectin-1, which is *β*-glucan receptor [48].

## 5. Effect of Mushroom Compounds on Immune Cells

Mushroom compounds injected directly into tumor cells or taken orally activate the immune cells to initiate a cell mediated or direct cytotoxicity on tumor cells after being recognized by pathogen recognition receptors. Compounds like lentinan increase the production of cytotoxic T lymphocytes and macrophages and also induce nonspecific immune responses [49].* Pleurotus tuber-regium* and* P. rhinoceros* extracts confer antitumor effects by promoting maturation of lymphocytes and NK cells and increasing macrophages proliferation, T helper cells, and CD4/CD8 ratio and population, which is accompanied by increase in weight and size of spleen, and this increase is attributed to the higher numbers of monocytes and granulocytes among other immune cells [32–34, 78]. Therefore, consumption of mushroom compounds initiates innate and adaptive immunity by enhancing immune-surveillance against cancer by involving monocytes, macrophages, NK cells, and B cells, CTLs secretion antitumor related cytokines and activation of immune organs, getting rid of cancers, and strengthening the weakened immune system [64, 65]. These actions by mushroom compounds lead to cancer cell apoptosis, cell cycle arrest, and prevention of angiogenesis and metastasis.

## 6. Inhibition of Proliferation and Cell Cycle Arrest by Mushroom Compounds

Various cancers, including hematological cancers in mouse and leukemia in humans, among other tumors are inhibited by mushrooms [27, 79]. Their mechanism of action is varied and is believed to include induction of apoptosis and upregulation of apoptosis inducing genes as well as arrest of cell division* in vitro and in vivo* [38, 39].

Mushroom compounds injected into tumor mass lead to apoptosis of the cells at different stages of cell cycle to curb tumor cell proliferation. For instance, lentinan and lectins from Shiitake are directly cytotoxic and cytostatic to MCF-7 breast cancer cells [53, 54]. They also show anti-inflammatory effect by reducing levels of neoangiogenic and granulocyte-chemoattractant factor IL-8 and increase infiltration of cytotoxic T cells by reducing intratumor formation of reactive oxygen and nitrogen species and ameliorating the skewed Th1/Th2 balance in late cancers [37, 40, 80–82]. This ability of phagocytes to infiltrate makes them important in eliminating advanced tumors by phagocytosis and secretion of cytokines for direct or indirect antitumor activities and antibody dependent cell mediated cytotoxicity (ADCC) [83]. Suppression of cell motility and blocking vasculature in tumor microenvironment is a good indicator for inhibition of cancer metastasis and proliferation.* Ganoderma lucidum* has the potential of suppressing cell motility, inhibiting cell proliferation, inducing apoptosis, and suppressing angiogenesis of highly invasive human breast and prostate cancer cells [43, 44]. PSK, on the other hand, when injected directly into human stomach tumors prior to surgery is quickly taken up by dendritic cells in and around the tumors, improving the survival and QoL of stomach cancer patients [50]. Thus, PSK has a direct cytotoxic effect on cancer cells. According to Hsu et al. [29], methanol extracts of* G. lucidum* and* G. tsugae* inhibit the growth of colorectal cancer cells within 72 hrs by downregulating cyclin A and B1 and upregulating p21 and p27, thereby arresting the cell cycle in G2/M, and thus they are able to suppress tumor growth, induce cell death, and inhibit cell proliferation in human colorectal cancer cells* in vivo*. Volman et al. [75] confirmed that there is modulation of the immune response of enterocytes, where extracts from mushrooms lower the transactivation of NF-kB in Caco-2 cells, with* A. blazei *Murill and* Coprinus comatus* having the pronounced decrease in NF-kB transactivation, which can cause tumor cells to stop proliferating, die, or become sensitive to the action of antitumor agents. In addition,* L. edodes* fruit body water extracts exhibit inhibitory effects on the proliferation of MCF-7 cells and DNA synthesis, indicating that the cytostatic effect of this mushroom extract is much potent on cell cycle of cancer cells [55]. MCF-7 cells treated with Huaier* (Trametes robiniophila)* extract show G0/G1 arrest leading to cell damage and apoptosis [22] and hot water extracts of* Coprinellus *sp.,* C. comatus*, and* Flammulina velutipes* have also shown inhibition of cellular proliferation of MCF-7, MDA-MB-231, and BT-20 cells [51].

Suffice it to say, researches have proven that mushrooms compounds exhibit anticancer potential in* in vitro*,* in vivo*, and clinical studies as summarized in Table 2. Therefore, critical research on anticancer mushroom compounds is important in the search for new drug discovery.

## 7. Conclusion and Future Perspective

Bioactive compounds from mushrooms have been shown to activate or modulate the immune system, thereby inhibiting cancer cell metastasis and growth. These compounds work by affecting the maturation, differentiation, and proliferation of immune cells. The major compounds of immune and cancer importance target the gut system, especially intestines as their site of contact and primary action, thereby affecting intestinal immunity and ultimately systemic immunity. These compounds are PAMP and act by interacting with receptors on leucocytes, upregulate genes associated with immunity, increase production of T lymphocytes and cytokines, activate activity of macrophages and cytokines, induce apoptosis, affect cell cycle, and increase infiltration of cytotoxic T cells in tumors. Critical studies on the mechanism of action and development of anticancer agents from mushrooms are very important so as to reduce the burden of cancer and improve quality of life of cancer patients.

Research, therefore, which targets modulation of the immune system to fight cancer, especially from mushroom compounds, is important. Future perspective should therefore be directed towards finding out the molecular mechanisms of different mushroom compounds in cancer immunotherapy and encouraging consumption of mushroom and other natural plant materials due to their holistic treatment. Further studies should be carried out on conservation of biodiversity of mushrooms, and critical analysis should be done to evaluate and compare the pharmacological importance and mushrooms of different regions.

## Figures and Tables

**Table 1:** Bioactive compounds from mushrooms with anticancer activity.

Mushroom	Cancer	Common name	Compound/extract
*Agaricus bisporus *	Breast, colorectal	White button	Polysaccharides, lectin
*Ganoderma lucidum *	Breast, colorectal, cervical, prostate, liver, and lung	Lingzhi/reishi	*Ganoderma* polysaccharides, polysaccharide-peptides
*Coriolus versicolor*	Breast, colorectal, and skin	Yun Zhi	Krestin, PSK, PSP
*Lentinus edodes*	Cervical/ovarian, gastric, and skin	Shiitake	Lentinan
*Grifola frondosa*	Breast and bladder	Maitake	Grifolan, Maitake D fraction
*Agaricus blazei*	Leukemia, hematological, stomach, and lung	Brazilian	*Agaricus* polysaccharides
*P. tuber-regium*	Liver	King tuber	Pleuran
*Flammulina velutipes *	Skin	Winter	Flammulin

Modified from Roupas et al. (2012).

**Table 2:** Summary of studies on the mechanism of action of mushrooms compounds.

Mushroom	Biological activity	Study	Reference
*Agaricus blazei *Murill (AbM)	Secretion of IL-8, TNF-*α*, and NO production by macrophages, inhibition of cancer cell growth, upregulation of expression and secretion of anticancer gene and cytokines IL-23, IL-12, IL-1, MCP-1, G-CSF, and TNF-*α*, apoptosis, and NK activation	*In vitro*	[25, 27, 36–40]
	Suppress tumor growth and inhibit angiogenesis, stimulate cytokine and leukocyte growth factors production, amelioration of skewed Th1/Th2 balance	*In vivo*	[26]

*Agaricus bisporus*	Induce apoptosis, inhibit angiogenesis, stimulate TNF-*α* production by BMM	*In vitro*	[27]

*Ganoderma lucidum*	Cytotoxic to cancer cells, inhibit cancer cell growth, stimulate T cells, upregulate expression and secretion of IL-1, IL-2, IL-6, TNF-*α*, and IFN*γ*, suppress cell motility and angiogenesis, inhibit proliferation and induce apoptosis, downregulate cyclins A and B and upregulate p21 and p27, arrest cell cycle	*In vitro*	[4, 14, 29, 41–44]

*Ganoderma tsugae*	Inhibit cancer cell growth, downregulate cyclins A and B and upregulate p21 and p27, arrest cell cycle	*In vitro*	[29]

*Grifola frondosa*	Activate macrophages, stimulate production of IL-1, IL-6, and IL-8, stimulate leukocytes	*In vitro*	[45–47]

*Sparassis crispa*	Augment immune system, enhance IL-8 synthesis, activate leukocytes	*In vitro*	[31]
	Stimulate splenocytes to secrete cytokines	*In vivo*	[48]

*Pleurotus tuber-regium*	Stimulate proliferation of NK cells, macrophages, and T cells	*In vitro*	[32–34]
	Maturation of lymphocytes, NK cells, and macrophages, increase weight and size of spleen	*In vivo*	[32–34]

*Polyporus rhinoceros*	Stimulate proliferation of NK cells, macrophages, and T cells	*In vitro*	[32–34]

*Schizophyllum commune*	Prolong life of head/neck/cervical cancer patients	Clinical	[30]

*Trametes robiniophila*	Apoptosis, antiangiogenesis, antimetastasis, drug resistance reversal, activation of immune system	clinical	[35]
	Apoptosis, G0/G1 cell cycle arrest, and Cell damage	*In vitro*	[22]

*Coriolus versicolor*	Invoke secretion of cytokines IL-1, IL-2, IL-6, IL-8, TNF-*α*, and TNF	*In vitro*	[49]
	Improve survival of stomach cancer patients	Clinical	[50]

*Coprinus comatus*	Inhibit cancer cell proliferation	*In vitro*	[51]

*Pseudallescheria boydii*	Stimulate secretion of IL-12 and TNF*α*	*In vivo*	[52]

*Coprinellus *sp.	Inhibit cancer cell proliferation	*In vitro*	[51]

*Lentinula edodes*	Stimulate secretion of IL-1, IL-2, IL-6, IL-8, TNF-*α*, and TNF, cytotoxic and cytostatic to breast cancer cells, inhibit proliferation cancer cells, inhibit DNA synthesis	*In vitro*	[49, 53–55]

*Flammulina velutipes*	Inhibit cancer cell proliferation	*In vitro*	[51]
